# Gastroesophageal reflux disease may causally associate with the increased atrial fibrillation risk: evidence from two-sample Mendelian randomization analyses

**DOI:** 10.3389/fcvm.2024.1393383

**Published:** 2024-06-03

**Authors:** Lei Wang, Yi Wei Lu

**Affiliations:** Cardiac Department, Aerospace Center Hospital, Beijing, China

**Keywords:** gastroesophageal reflux disease, atrial fibrillation, Mendelian randomization, genome-wide association study, single nucleotide polymorphisms

## Abstract

**Background:**

The risk of atrial fibrillation (AF) is increased in individuals with gastroesophageal reflux disease (GERD), according to observational research. The causal significance of this association is still unclear. This study sought to assess GERD's role as a potential contributing factor in AF.

**Methods:**

With the use of a two-sample Mendelian randomization (MR) technique, we assessed the causal relationship between GERD and AF. The association of genetic variants with GERD was examined using data from a recent genome-wide association study (GWAS) that included 602,604 people. Data on the association between genetic variations and AF was obtained from a second GWAS with 1,030,836 participants. The effect sizes were examined based on the inverse-variance weighted method. Additional statistical techniques, including MR-Egger, simple mode, weighted mode, MR Pleiotropy Residual Sum, outlier, and weighted median were used in the sensitivity analysis.

**Results:**

MR analyses in inverse-variance weighted models, using 76 single nucleotide polymorphisms (SNPs) as markers, revealed a relationship between genetically predicted GERD and a greater AF incidence [odds ratio (OR): 1.165, 95% CI 1.102–1.231; *P* = 7.637 × 10^−8^]. According to MR-Egger, there was no evidence of gene pleiotropy that could be found (intercept = 0.003, *P* = 0.581). The findings of the sensitivity study, which used several MR methods, were found to be reliable.

**Conclusion:**

The MR analysis revealed a correlation between GERD and increased AF incidence, supporting the idea that treating patients with GERD as early as possible might reduce their chance of developing AF.

## Background

Presently, 2%–3% of population all around the world are affected by atrial fibrillation (AF). The electrical signals of AF in the atria are rapid and disorganized producing an irregular heartbeat (1). Smoking, cardiac surgery, sleep apnea, obesity, thyroid disease, valvular heart disease, and chronic renal disease are risk factors that increase the risk of developing AF. In addition to a higher risk of heart failure and stroke, those with AF may experience fatigue, dyspnea, dizziness, and heart palpitations (1). AF can contribute to considerable societal and medical expenses with major health and economic consequences.

Gastroesophageal reflux disease (GERD) is a condition that affects the digestive tract and is a prevalent presenting symptom. GERD occurs when acidic stomach juices, fluids, and food are backed up from the stomach into the esophagus, causing extreme discomfort. GERD and its correlation with atrial fibrillation (AF) are being studied (2). There is likely a link between GERD and AF due to their shared risk factors, including sleep apnea, obesity, and local inflammation that worsens with age (2). The prospective observational studies and retrospective database analysis suggest an association between GERD and AF (3–6). A systematic review showed the summary-adjusted relative risk for GERD-induced AF was 1.06 (95% CI, 0.86–1.31) (7). The studies also demonstrated that proton pump inhibitors may reduce the burden of symptomatic arrhythmia (8). However, an objective evaluation of GERD showed a temporal correlation between episodes of reflux and cardiac arrhythmia is low (9). Whether GERD performs a casual function in the development of AF is uncertain since the link between the two has not been extensively explored due to the possibility of biases such as confounding or reverse causality (10).

Mendelian randomization (MR) is a strategy that employs instrumental variable (IV) approaches to determine the causative relationship between complex human characteristics and genetic risk factors (11, 12). MR Studies can determine the causal relationship between exposure and disease outcomes by removing unmeasured confounders and reverse causality since IVs exposed to random allocation before conception are not expected to be impacted by disease status. Variants that influence GERD can also, to some degree, have some bearing on AF if a risk factor, such as GERD, may have a direct impact on an outcome, such as AF. Nonetheless, due to the presence of horizontal pleiotropy, any other potential pathways for this variant's influence on AF must be ruled out (13). In this investigation, we aimed to conduct an MR study to verify the causative effect of GERD on the pathogenesis of AF from a major genome-wide association study (GWAS) (14).

## Methods

**Figure 1:**
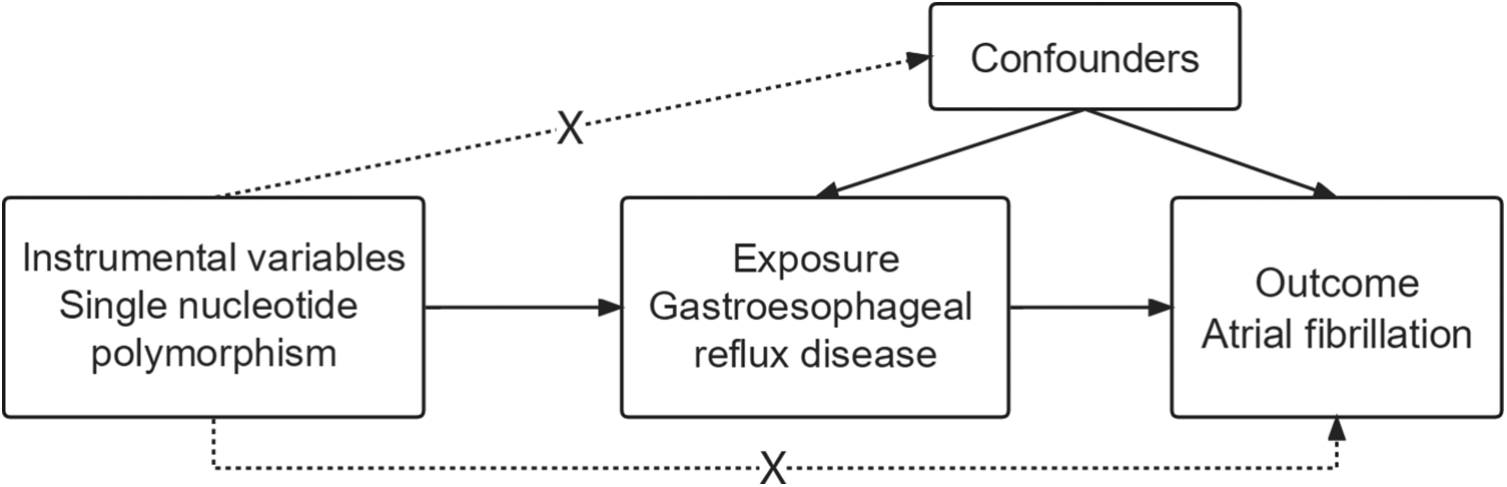
Flowchart depicting the analyses and core hypotheses for a two-sample Mendelian randomization (MR) study. Prediction of the association between GERD and the risk of atrial fibrillation in an MR model. The strategy assumes that GERD-related genetic variations have an effect via the condition itself and are unrelated to confounders.

The two-sample MR was conducted using published pooled data on characteristics of interest from major European individuals from GWAS (11, 13). Each cohort participating in the GWAS was ethically approved and agreed upon by the participants and provided summary-level information for analysis. In particular, The two-sample MR approach was used to examine the possible causal effect that GERD characteristics have on AF (Figure 1).

### GWAS summary data for GERD

An extensive European GWAS Meta-analysis was conducted to search for genes associated with GERD. The study included 602,604 individuals (129,080 patients with GERD and 473,524 controls) (15). A broader definition of GERD was used in the study since it relied on patient reports, physician assessments, and examination of medical records. A total of 80 single-nucleotide polymorphisms (SNPs) were found to have a significant association with GERD (*p* < 5 × 10^−8^), and the prerequisite for using a two-sample MR approach is that the instrument is devoid of any linkage disequilibrium (LD) (r^2^ < 0.001, distance threshold >10,000 kb). A detailed illustration of the 80 SNPs may be found in Supplementary Table S1. Notably, these 80 SNPs accounted for 0.51% points of the variance in GERD (16).

### Summary data from the GWAS for AF

Only summary data from Europeans were utilized in this study so that we could avoid demographic heterogeneity. A second large European GWAS Meta-analysis was mined for AF genes; it included information from 1,030,836 people, of whom 60,620 with AF and 970,216 acted as controls (16). Six studies consisting of participants of European ancestry made up the sample (the Nord-Trøndelag Health Study [HUNT], deCODE, Michigan Genomics Initiative [MGI], DiscovEHR, UK Biobank, and AFGen Consortium). Cases and controls having genotype data were identified using a mix of inpatient, outpatient, and emergency department discharge diagnoses (ICD-9 and ICD-10). There were 142 genes and 111 genomic areas discovered to have independent risk mutations, and 151 functional candidate genes were ranked according to their probable involvement in AF. Genotyping data, imputation, and quality assurance are among the additional resources available (16).

### Selection of genetic instrumental variables

When an individual exposure SNPS is missing from the final data, it is not replaced with a proxy SNPS using LD tagging. Summary data for each of the 80 SNPs linked to GERD were collected from the GWAS Meta-analysis. It is crucial to determine if the effect of SNPS on exposure and the influence of alleles on outcomes are consistent in two-sample MR. The two GWAS findings’ SNPs were consistent in terms of allele frequency. SNPs were omitted if there was inconsistency. Palindromicity and the presence of frequent intermediate alleles led to the elimination of three of the 80 SNPs (rs2145318, rs2358016, and rs957345).

The underpinning outliers were filtered out before MR analysis utilizing Mendelian randomized multiplicity residual sums and outliers (MR-PRESSO) tests, which are most useful upon observing horizontal pleiotropy <50% of the instruments (17). In the MR-PRESSO analysis, one outlier (rs9940128) was identified (*p* = 0.662). Finally, 76 SNPs were involved in the MR analysis.

Owing to the MR analysis's use of overlapping sample individuals to ascertain the heritability of exposure and outcomes, a slight increment in the weak instrumental bias is possible. We used the F-statistic of SNP to determine how significant the exposure IVs were (18). The F-statistic of 38 for the instruments was significantly greater than the average of 10, indicating excellent prediction power for GERD (19). To determine R2, the following formula was utilized: R^2 ^= [2 × EAF (1- EAF) × β^2^], whereby EAF denotes the effect of allele frequency and β denotes the presumed genetic impact on GERD. We calculated the F-statistic, based on the following formula, to rank the significance of each SNP: F = [R^2^ × (n−1−k)]/ [(1−R^2^) × k], where k is the total count of SNPs, *n* is the sample size, and R^2^ indicates the proportion of phenotypic variation that can be attributable to underlying genetic variants (20).

### Testing Mendelian randomization assumptions

Three fundamental presumptions must be true for MR studies: (1) Genetic variants and exposure have a strong correlation; (2) The genetic variants and potential confounders were not linked; (3) Apart from the exposure modality, genetic variation had no effect on the outcomes (Figure 1). The inclusion of unidentified possible confounders makes it challenging to test hypotheses 2 and 3. Therefore, to ascertain if there is horizontal pleiotropy—whether hereditary variables impact AF in addition to GERD characteristics—we computed the regression coefficient of MR Egger and assessed the significant intercept.

### Statistical analyses

We divided the Beta of the associated SNPS in the outcome dataset by the Beta of the same SNPS in the exposure dataset to get the Wald ratio and evaluate the causal effect of each IV. To determine the direction of any possible causation between GERD and AF risk, we conducted an MR-Steiger analysis. We used a multiplicative inverse-variance weighted (IVW) model in the case of significant Cochran's Q value (*P* < 0.05), and heterogeneity is acceptable (21). We applied a fixed-effects model in every other scenario. In the primary study, the characteristics linked to genetically predicted exposures and the risk of AF were compared using the IVW approach. Wald ratios for every SNP were analyzed in a meta-analysis. The foundation of IVW was the assumption that the findings could only have been affected by the exposure of interest.

The findings of single and multi-SNP studies are merged and displayed using scatter and forest plots. The 95% confidence intervals for single-SNP and multi-SNP impact estimates are displayed next to one another in a graphic that resembles a forest plot. Regression lines determined by multiple SNP analyses were added to the scatter plot to compare the effects of a single SNPS on exposure and outcomes (having matching standard deviations in either direction).

We estimate that our MR analysis, with a sample size of 602,604 and an alpha of 5%, will detect an OR of 1.10 for AF per odds of GERD (22). The analyses were completed utilizing Two-Sample MR (v0.5.6) (23) and MRPRESSO (v1.0) in R (v4.1.2). The strength of the association between a projected genetic risk for GERD and the risk of AF is described using ORs with 95% CIs. We concluded that the causal results of multiple MR methods were consistent in direction and magnitude (see below) and passed nominal significance in IVW. It was determined that *p* < 6.579 × 10^−4^ (0.05/76) provided statistically significant proof of a causal relationship. *P*-values < 0.05 but above the Bonferroni correction threshold were considered suggestive evidence of potential causality.

### Sensitivity analysis

To gauge the connections’ robustness, we conducted multiple sensitivity tests. Initially, we employed a weighted median approach to examine the linkages, assuming that at least 50% of the weights came from reliable instruments (24). Additionally, simple model, MR-Egger, and weighted model approaches are employed even though they are less effective since they may offer more precise estimates in a broader variety of scenarios (with a wider CI). Additionally, we performed a leave-one analysis based on the IVW MR technique, where each SNP was omitted from the analysis separately, to assess the probable effects of outliers and/or pleiotropy SNPS. Moreover, a funnel plot was applied to investigate heterogeneity.

## Results

To determine whether the causal effect estimates were robust, we conducted a Steiger-MR analysis. According to Steiger-MR, SNPs account for greater variation in exposure than in outcome (*P* = 0.725). Because Cochran's Q value was insignificant (*P* = 0.092), the relationship between genetically determined GERD-related characteristics and the risk of AF was evaluated utilizing the fixed-effects IVW method. As indicated in Table 1, we found evidence of a probable causal relationship between GERD and AF in traditional MR analysis using the IVW approach [odds ratio (OR): 1.165, 95% CI 1.102–1.231; *P* = 7.637 × 10^−8^]. The causal estimates made from each IV were shown on the forest plot (Supplementary Figure S1) and scatter plot (Supplementary Figure S2).

**Table 1:** Association between GERD and AF by Mendelian randomization models.

Method	nsnp	OR	95%CI	*P*-Value
Inverse variance weighted	76	1.165	1.102–1.231	7.637 × 10^−8^
Weighted median	76	1.201	1.112–1.297	3.032 × 10^−6^
MR Egger	76	1.066	0.775–1.465	0.695
Simple mode	76	1.317	1.045–1.661	0.022
Weighted mode	76	1.299	1.075–1.569	0.008

OR, odds ratio; MR, Mendelian randomization; SNP, single nucleotide polymorphism; CI, confidence intervals.

We discovered that GERD and AF exhibited a causal relationship utilizing the weighted median model (OR = 1.201, 95% CI = 1.112–1.297; *P* = 3.032 × 10^−6^). Both weighted mode and simple mode techniques produced consistent results (*P* < 0.05).

No evidence of pleiotropy was observed (MR-Egger regression test, intercept = 0.003, *P* = 0.581). The leave-one-out analysis illustrated that none of the instruments could independently account for the overall effect of GERD on AF (Supplementary Figure S3). According to the IVW and MR Egger model, no statistically significant heterogeneity was observed in the causal estimates among IVs (Supplementary Figure S4).

## Discussion

Our two-sample MR analysis was based on data from publically accessible GWASs to probe the link between GERD and AF risk and draw inferences regarding their causality. Genetic susceptibility to GERD was associated with an increased likelihood of developing AF, as shown by the statistical causal relationship.

Previous observational studies did correlate GERD with AF, but their findings were contradictory. Patients with GERD, and specifically those with more severe GERD-associated symptoms, were shown to have a higher chance of developing AF as opposed to those without the condition as per the findings of the epidemiological data obtained from these observational studies. However, these studies did not provide enough evidence to prove that GERD exhibits a causal relationship with AF (25). The presence of AF was associated with an increased incidence of GERD in an observational study, suggesting that this condition might be a risk factor for its development (26). Nonetheless, the causality between GERD and AF is uncertain because of the possibility of bias in observational studies related to several confounders. It is difficult to use conventional epidemiological methodologies to determine the causal relationship between GERD and AF. Moreover, we adopted a two-sample MR, a technique that accounts for the effects of potential confounders by employing genetic variations with a biological pathway separate from the exposure of relevance, in contrast to these conventional epidemiological investigations. Our findings held up across multiple sensitivity studies that probed the effect of pleiotropy on causation estimations. There is presently insufficient data to decide on anti-GERD treatment schemes for AF patients (27). These findings need to be confirmed by further research methods, such as large-scale intervention trials and prospective cohort studies.

Based on the results of our two-sample MR study, we infer that GERD may increase the risk of AF. Multiple potential biological mechanisms have been hypothesized to account for the beneficial impact of GERD on AF. Previous research has shown that proinflammatory cytokines play a role in the development of GERD (25, 28). Injuries to the esophagus may cause the release of cytokines locally, creating conditions favorable to the development of AF. Inflammatory cells have been observed infiltrating the atriums of patients with AF, indicating an inflammation-AF link (29–31). Furthermore, GERD can cause autonomic nervous system dysfunction (32–34), consequently increasing AF risk (35). To help researchers and physicians develop new preventive and therapeutic methods, further analysis into the molecular mechanisms behind these relationships is required.

### Limitation

Some drawbacks are present in this MR study. First, because this information was generated from GWAS data of European ancestry, it must be confirmed in other ethnic groups to guarantee that it is relevant to people of other racial backgrounds. Second, the results from the research cannot differentiate between AF episodes (including atrial flutter and paroxysmal, persistent, and chronic AF), disorders that may have different etiologies (36, 37). Third, it was difficult to determine the extent of overlapping between the outcome and the exposure samples since sample and diagnostic data were unavailable.

## Conclusion

A higher incidence of AF was shown to be associated with GERD, as determined by the MR analysis, lending support to the idea that treating GERD patients early might reduce their risk of developing AF.

## Data Availability

Publicly available datasets were analyzed in this study. This data can be found here: The datasets generated and/or analysed during the current study are available in the IEU GWAS repository (https://gwas.mrcieu.ac.uk).
