# Supplementary material for: Gastroesophageal reflux disease may causally associate with the increased atrial fibrillation risk: evidence from two-sample Mendelian randomization analyses
Source: Front Cardiovasc Med. 2024 Jun 3;11:1393383. doi: 10.3389/fcvm.2024.1393383 (PMC11182450; doi:10.3389/fcvm.2024.1393383)
Supplement: Supplementary file 1 [file Datasheet1.doc]

Supplemental Material

| SNP | Effect_allele | Other_allele | Chr | Position | EAF | beta | se |
| --- | --- | --- | --- | --- | --- | --- | --- |
| rs17379561 | T | A | 1 | 98340139 | 0.144391 | 0.0530714 | 0.0093 |
| rs1937450 | G | T | 1 | 66478840 | 0.537739 | 0.0315845 | 0.0067 |
| rs2782641 | A | G | 1 | 44013355 | 0.612669 | 0.0270882 | 0.0068 |
| rs2815749 | G | A | 1 | 72814783 | 0.800974 | 0.0388767 | 0.0084 |
| rs3766823 | A | G | 1 | 32197257 | 0.171467 | 0.0393599 | 0.0088 |
| rs569356 | G | A | 1 | 29136686 | 0.140835 | -0.037919 | 0.0099 |
| rs7527682 | G | A | 1 | 189172684 | 0.53725 | -0.026684 | 0.0067 |
| rs7541875 | G | A | 1 | 190957589 | 0.426069 | 0.0273972 | 0.0067 |
| rs903678 | A | G | 1 | 201809918 | 0.339412 | 0.0277378 | 0.007 |
| rs1011407 | G | A | 2 | 60665768 | 0.121628 | -0.0420618 | 0.0104 |
| rs12997558 | A | G | 2 | 41704580 | 0.358751 | 0.0278184 | 0.0069 |
| rs13409451 | G | A | 2 | 144257639 | 0.392403 | -0.0277081 | 0.0069 |
| rs1596747 | G | A | 2 | 193802478 | 0.494136 | 0.0310869 | 0.0066 |
| rs2358016 | G | C | 2 | 162007430 | 0.498113 | 0.028271 | 0.0066 |
| rs4300861 | T | C | 2 | 22549441 | 0.38208 | 0.0307132 | 0.0069 |
| rs6711584 | A | G | 2 | 104421692 | 0.452019 | 0.0322545 | 0.0067 |
| rs6722661 | A | G | 2 | 100806588 | 0.364669 | -0.0322543 | 0.007 |
| rs7600261 | T | C | 2 | 212622818 | 0.306391 | 0.0338034 | 0.0073 |
| rs2016933 | G | C | 3 | 65653157 | 0.730053 | -0.0310253 | 0.0074 |
| rs2240326 | A | G | 3 | 50128386 | 0.473775 | -0.0471681 | 0.0066 |
| rs6780459 | T | A | 3 | 104624105 | 0.746622 | 0.0305507 | 0.0077 |
| rs7612999 | A | G | 3 | 35678337 | 0.245338 | 0.0305231 | 0.0076 |
| rs10010963 | T | C | 4 | 159839313 | 0.616433 | -0.0269803 | 0.0068 |
| rs13107325 | T | C | 4 | 103188709 | 0.074445 | 0.0701443 | 0.0142 |
| rs1510719 | C | T | 4 | 140938116 | 0.383439 | -0.0388844 | 0.0068 |
| rs2164300 | T | C | 4 | 67813017 | 0.523279 | -0.0264751 | 0.0067 |
| rs7675588 | A | C | 4 | 80734978 | 0.794635 | -0.0335228 | 0.0081 |
| rs7685686 | G | A | 4 | 3207142 | 0.422353 | -0.0279222 | 0.0067 |
| rs11953061 | T | C | 5 | 120144025 | 0.338908 | 0.0281599 | 0.007 |
| rs1592757 | C | G | 5 | 103889998 | 0.355772 | 0.0311051 | 0.007 |
| rs329122 | A | G | 5 | 133864599 | 0.419631 | -0.0289529 | 0.0067 |
| rs12204714 | T | C | 6 | 152235339 | 0.632223 | -0.028817 | 0.0069 |
| rs2145318 | A | T | 6 | 26496603 | 0.486642 | 0.0353431 | 0.0067 |
| rs2744961 | T | C | 6 | 34655000 | 0.358437 | 0.0292007 | 0.007 |
| rs3828917 | T | G | 6 | 31465917 | 0.041826 | 0.0671113 | 0.0187 |
| rs4713692 | T | C | 6 | 33807638 | 0.367808 | -0.0276128 | 0.007 |
| rs9372625 | A | G | 6 | 98344031 | 0.383042 | -0.037727 | 0.0069 |
| rs9373363 | G | A | 6 | 143150043 | 0.253631 | -0.0326836 | 0.0076 |
| rs9396740 | A | G | 6 | 17023108 | 0.248794 | -0.031493 | 0.0077 |
| rs11762636 | A | C | 7 | 2061111 | 0.180282 | -0.0514827 | 0.0086 |
| rs2043539 | A | G | 7 | 12253880 | 0.41866 | 0.0272058 | 0.0066 |
| rs2106353 | T | G | 7 | 126506598 | 0.231451 | 0.0367491 | 0.008 |
| rs215614 | A | G | 7 | 32347335 | 0.629725 | -0.0328541 | 0.0068 |
| rs2396133 | G | A | 7 | 109197067 | 0.475329 | 0.0293547 | 0.0067 |
| rs2396766 | A | G | 7 | 114318071 | 0.47308 | 0.0322057 | 0.0067 |
| rs3863241 | T | C | 8 | 73890335 | 0.52696 | 0.0324982 | 0.0067 |
| rs903959 | A | T | 8 | 142630782 | 0.399262 | 0.0291631 | 0.0068 |
| rs3793577 | G | A | 9 | 23737627 | 0.538279 | 0.0270309 | 0.0067 |
| rs4382592 | G | T | 9 | 134870755 | 0.699524 | -0.0302679 | 0.0072 |
| rs7032155 | A | C | 9 | 122672771 | 0.59185 | 0.02775 | 0.0068 |
| rs1021363 | G | A | 10 | 106610839 | 0.641992 | -0.031217 | 0.007 |
| rs12357321 | A | G | 10 | 21790476 | 0.311087 | 0.0317159 | 0.0073 |
| rs761777 | G | A | 10 | 134938075 | 0.254034 | 0.0345341 | 0.0083 |
| rs10837002 | G | C | 11 | 38565727 | 0.35122 | 0.0276491 | 0.007 |
| rs2734839 | T | C | 11 | 113286490 | 0.606693 | -0.0283478 | 0.0068 |
| rs7942368 | T | C | 11 | 76465362 | 0.214659 | -0.0339683 | 0.008 |
| rs1479405 | T | C | 12 | 15387519 | 0.3217 | 0.0314843 | 0.007 |
| rs1716171 | T | C | 12 | 123716376 | 0.790024 | 0.0383981 | 0.0081 |
| rs324769 | T | C | 12 | 83969240 | 0.449179 | -0.0267699 | 0.0067 |
| rs773109 | A | G | 12 | 56374695 | 0.335269 | -0.0380572 | 0.0071 |
| rs1334297 | A | G | 13 | 58335375 | 0.734249 | -0.0387984 | 0.0075 |
| rs9517313 | C | G | 13 | 99105892 | 0.383217 | 0.0331144 | 0.0068 |
| rs9529055 | A | G | 13 | 66957533 | 0.475633 | 0.0266604 | 0.0067 |
| rs9542729 | G | C | 13 | 31833578 | 0.20244 | -0.0363194 | 0.0083 |
| rs10133111 | A | G | 14 | 103377321 | 0.162996 | 0.0417875 | 0.0089 |
| rs942065 | A | G | 14 | 94032065 | 0.634045 | 0.0307384 | 0.007 |
| rs957345 | G | C | 14 | 75276079 | 0.540235 | 0.0290528 | 0.0066 |
| rs12598916 | G | C | 16 | 60658751 | 0.274798 | -0.0332614 | 0.0074 |
| rs7206608 | G | C | 16 | 82872628 | 0.322927 | 0.0291541 | 0.0071 |
| rs9940128 | A | G | 16 | 53800754 | 0.421755 | 0.0332513 | 0.0067 |
| rs12453010 | T | C | 17 | 50316131 | 0.394803 | 0.0296967 | 0.0068 |
| rs12967855 | G | A | 18 | 35138245 | 0.670435 | -0.0365451 | 0.0071 |
| rs1431196 | G | A | 18 | 50832102 | 0.428432 | 0.0324197 | 0.0067 |
| rs7241572 | A | G | 18 | 77580712 | 0.209101 | 0.0365511 | 0.0085 |
| rs2023878 | T | C | 19 | 18834124 | 0.192377 | -0.0362846 | 0.0084 |
| rs9636202 | A | G | 19 | 18449238 | 0.26663 | -0.035044 | 0.0077 |
| rs1883842 | G | T | 20 | 41223062 | 0.279255 | 0.0308332 | 0.0075 |
| rs2834005 | C | T | 21 | 34291708 | 0.315 | 0.0296997 | 0.0073 |
| rs2838771 | C | G | 21 | 46501576 | 0.646721 | -0.0280984 | 0.0071 |
| rs9615905 | T | C | 22 | 48875699 | 0.458193 | 0.0275657 | 0.0068 |

Table S1. The information on the 80 selected SNPs with gastroesophageal reflux disease.

Abbreviations: SNP single nucleotide polymorphism, Chr chromosome, EAF effect allele frequency, se standard error.


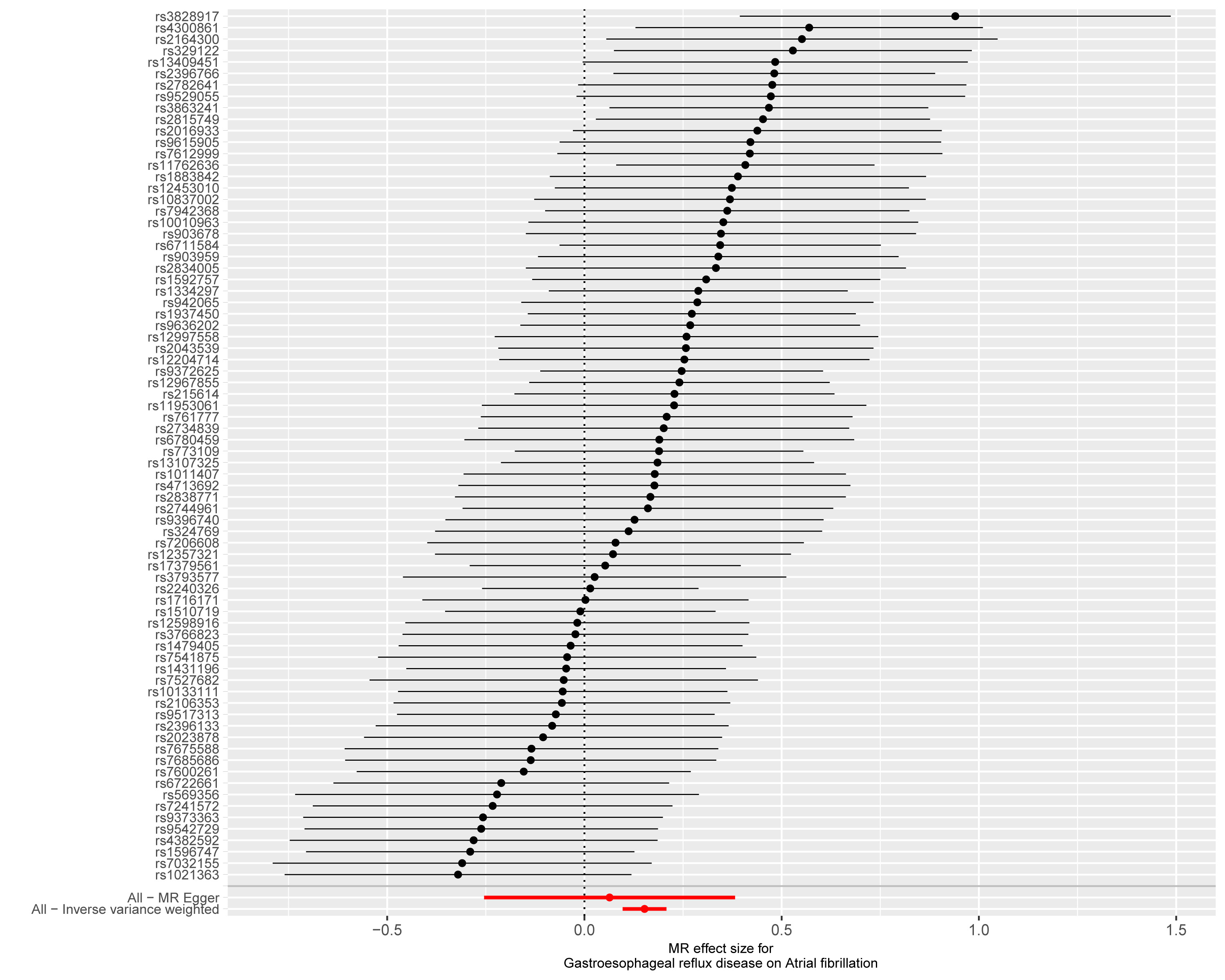


Figure S1: The forest plot of Mendelian Randomization analyses

Mendelian Randomization effect size for gastroesophageal reflux disease on atrial fibrillation for individual variants, MR-Egger and Inverse Variance Weighted.


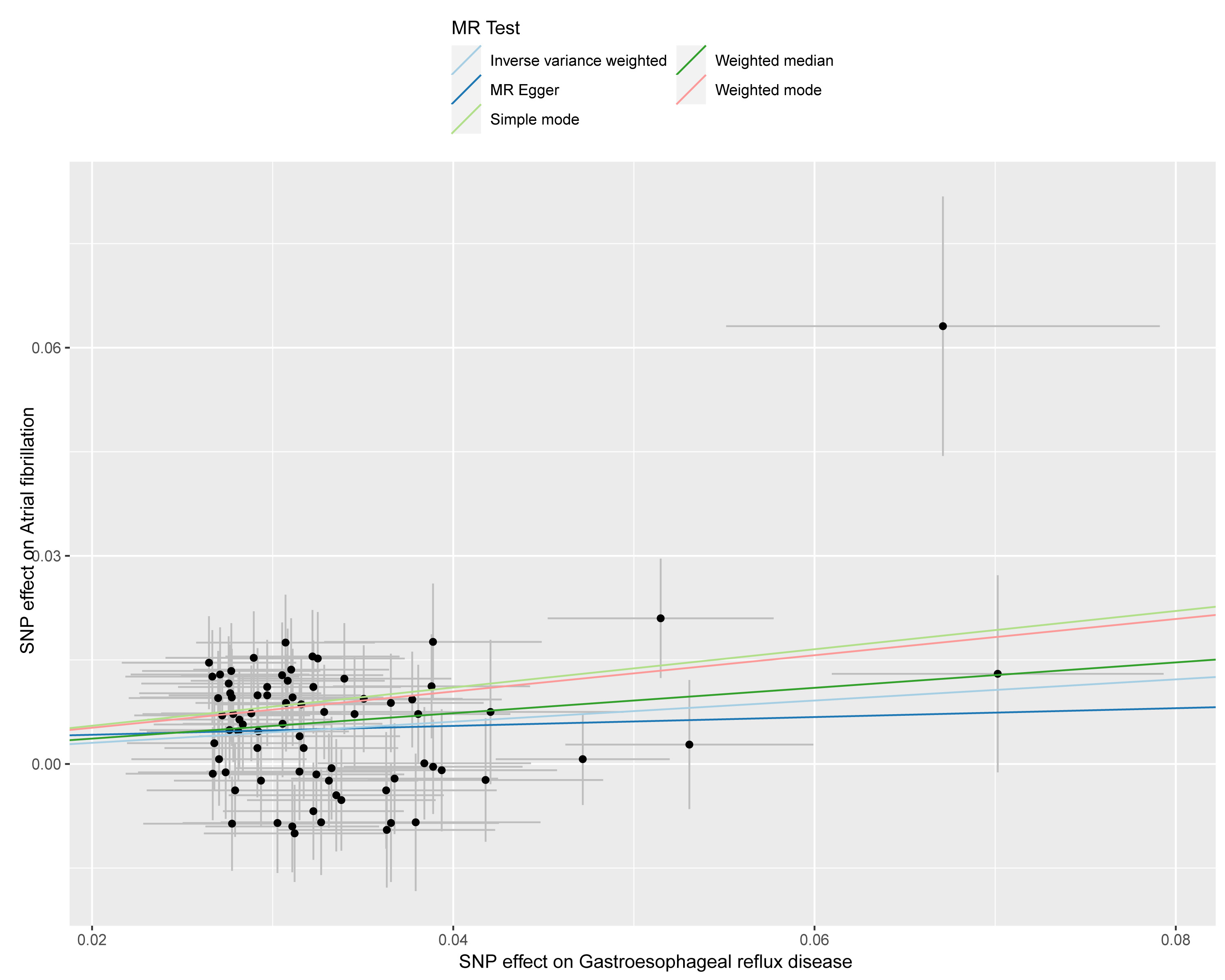


Figure S2: The scatter plot of Mendelian Randomization analyses


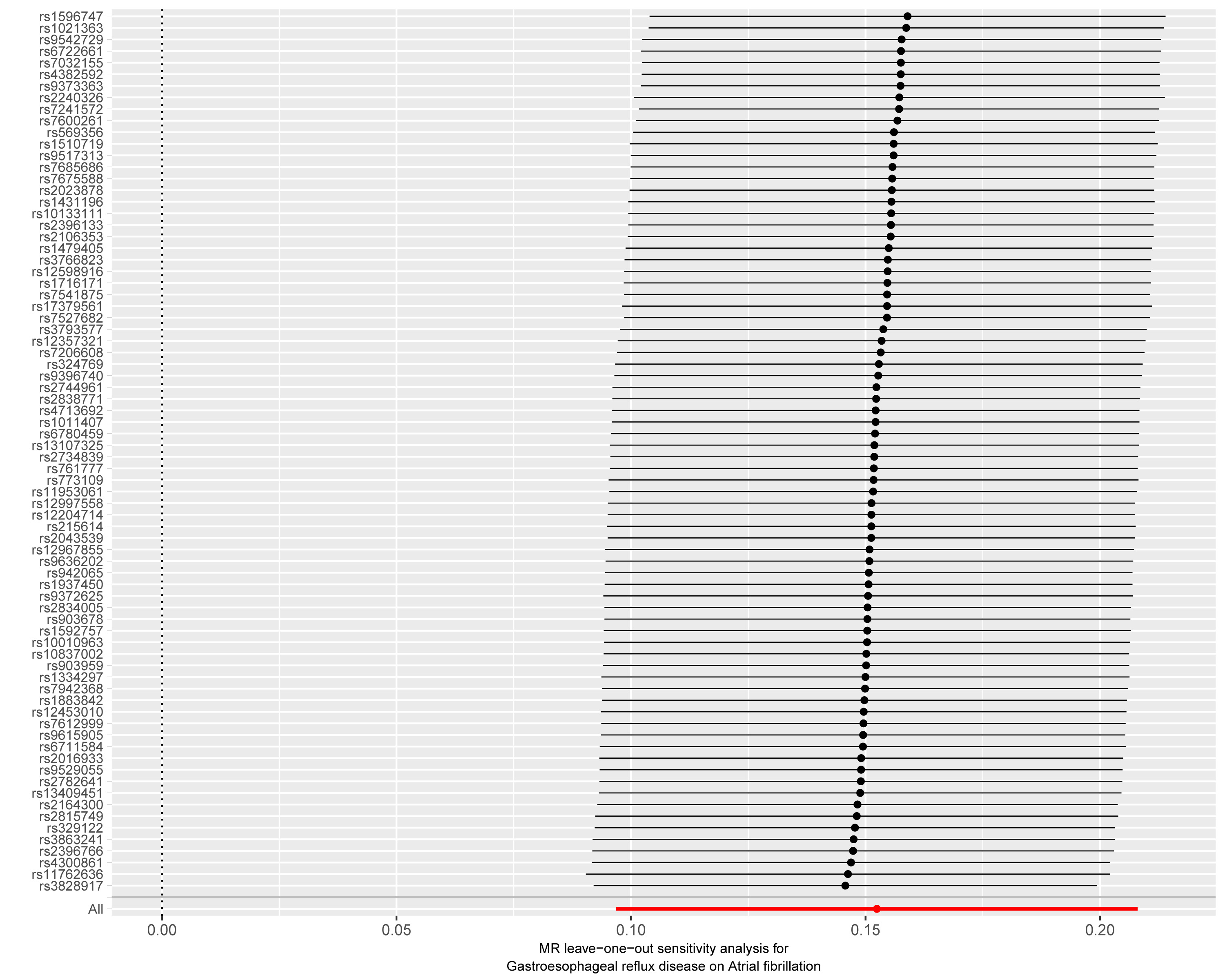


Figure S3: Mendelian Randomization leave-one-out analyses


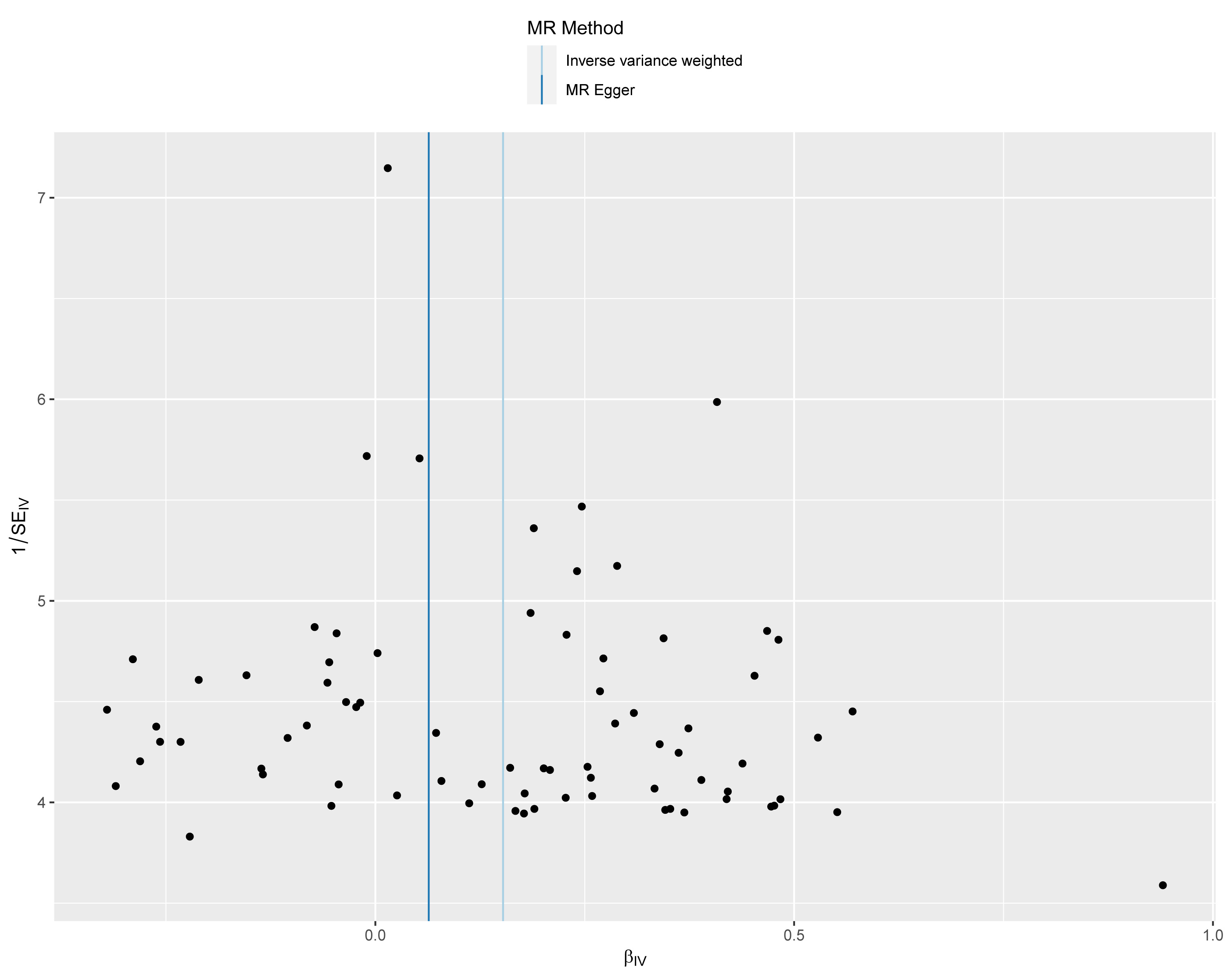


Figure S4: The funnel plot of Mendelian Randomization analyses
